# Investigation of the Corrosion Behavior of Atomic Layer Deposited Al_2_O_3_/TiO_2_ Nanolaminate Thin Films on Copper in 0.1 M NaCl

**DOI:** 10.3390/ma12040672

**Published:** 2019-02-24

**Authors:** Michael A. Fusco, Christopher J. Oldham, Gregory N. Parsons

**Affiliations:** Department of Chemical and Biomolecular Engineering, North Carolina State University, Raleigh, NC 27695, USA; mfusco@ncsu.edu (M.A.F.); cjoldham@ncsu.edu (C.J.O.)

**Keywords:** atomic layer deposition, corrosion protection, copper, aluminum oxide, titanium oxide, nanolaminate, electrochemical impedance spectroscopy, barrier coatings

## Abstract

Fifty nanometers of Al_2_O_3_ and TiO_2_ nanolaminate thin films deposited by atomic layer deposition (ALD) were investigated for protection of copper in 0.1 M NaCl using electrochemical techniques. Coated samples showed increases in polarization resistance over uncoated copper, up to 12 MΩ-cm^2^_,_ as measured by impedance spectroscopy. Over a 72-h immersion period, impedance of the titania-heavy films was found to be the most stable, as the alumina films experienced degradation after less than 24 h, regardless of the presence of dissolved oxygen. A film comprised of alternating Al_2_O_3_ and TiO_2_ layers of 5 nm each (referenced as ATx5), was determined to be the best corrosion barrier of the films tested based on impedance spectroscopy measurements over 72 h and equivalent circuit modeling. Dissolved oxygen had a minimal effect on ALD film stability, and increasing the deposition temperature from 150 °C to 250 °C, although useful for increasing film quality, was found to be counterproductive for long-term corrosion protection. Implications of ALD film aging and copper-based surface film formation during immersion and testing are also discussed briefly. The results presented here demonstrate the potential for ultra-thin corrosion barrier coatings, especially for high aspect ratios and component interiors, for which ALD is uniquely suited.

## 1. Introduction

Copper is ubiquitous in numerous industries due to its relatively low cost coupled with its malleability and high electrical and thermal conductivities. It has uses in everything from residential and commercial plumbing [1,2,3,4] and electrical wiring to industrial heat exchangers [5,6,7] and high-powered electronics [8,9,10,11]. Copper possesses adequate aqueous corrosion resistance due to the formation of a semi-protective native oxide film. Still, it corrodes at a finite rate and is susceptible to pitting dependent on solution constituents, pH, and temperature [2,3,4,12,13,14,15,16].

One specific application of interest here is the use of copper in radio frequency (RF) devices (i.e., traveling-wave tubes and crossed-field amplifiers). These devices typically contain fluid-cooled copper collectors and/or copper cooling channels [17,18,19,20,21,22,23,24]. Often, the cooling fluid is specified as deionized (DI) water with low dissolved oxygen content, in which case corrosion of the wetted copper components is insignificant within the useful lifetime of the device. However, the presence of contaminants—such as chlorides, additional oxygen, and carbon dioxide—in the cooling water supply can accelerate the corrosion to unacceptable levels. Chlorides are known to be particularly aggressive toward copper [12,14,25], as with many other metals, and are common contaminants in water supplies. Copper readily forms salt compounds in the presence of chlorides and can have its protective oxide film locally disrupted, allowing for pitting to occur.

The corrosion of copper in chloride media has been thoroughly studied [13,14,15,26,27,28,29,30], from which it has been concluded that the anodic and cathodic half reactions are given by Equations (1) and (2), respectively. The anodic reaction proceeds in two steps with the mostly insoluble CuCl produced in the first step and soluble CuCl_2_^−^ produced in the second. CuCl builds up as a film on the Cu surface, leading to passivation, or, more appropriately, pseudo-passivation. Higher chloride concentrations tend to shift the equilibrium of Equation (1) toward CuCl_2_^−^ and can even produce higher chloride complexes (i.e., CuCl_3_^2−^ and CuCl_4_^3−^) [27,31]. This increases dissolution of the CuCl surface film and accounts for the more aggressive corrosion seen in solutions with higher chloride content. Additionally, cuprous oxide (Cu_2_O) may be produced from CuCl_2_^−^, but its stability also decreases as the chloride content increases [31].

Oxygen reduction and water reduction (Equation (2)) dominate the cathodic current, though there are complications in the presence of copper corrosion products and surface films [31], including the reduction of CuCl on the copper surface [30]. Water reduction dominates when dissolved oxygen levels are low, whereas oxygen reduction produces high cathodic currents in oxygenated solutions [32]. Although the corrosion of copper in pure water via water reduction and hydrogen evolution in the absence of oxygen is still under debate [33,34,35,36,37], it is reasonable to expect that this reaction could play a role in the presence of chlorides and at sufficient overpotential.

(1) $$\begin{array}{l} Cu+Cl^{-}\to CuCl+e^{-}\\ CuCl+Cl^{-}\to CuCl_{2}^{-} \end{array}$$

(2) $$\begin{array}{l} O_{2}+2H_{2}O+4e^{-}\to 4OH^{-}\\ 2H_{2}O+2e^{-}\to H_{2}+2OH^{-} \end{array}$$

A common approach to improve the corrosion resistance of copper is to alloy it with other metals, such as aluminum and nickel. However, alloying is not always an option, as it can significantly reduce the electrical (and thermal) conductivity [38]. Another option is the application of barrier coatings to the copper surface. To preserve the desirable bulk properties of the copper, the barrier coatings should be as thin as possible. Additionally, copper components of RF devices are often of complex geometry or high aspect ratio, which narrows the available thin film deposition techniques.

Atomic layer deposition (ALD) is a vapor-phase technique that is uniquely suited for deposition of conformal thin films over complex, non-planar surfaces and through high-aspect-ratio structures, like tubing. ALD has no line of sight requirement with sub-nanometer thickness control [39,40,41,42,43]. ALD can also proceed at low temperatures (<150 °C) [44,45], avoiding temperature limits of fabricated components and even enabling deposition on thermally-sensitive substrates, such as polymers [46,47,48,49,50] and microelectronics [51,52].

Alumina (Al_2_O_3_) and titania (TiO_2_) are two of the most widely-studied ALD processes. Deposition of alumina via trimethylaluminum (TMA, Al(CH_3_)_3_) and H_2_O is thermodynamically favorable and nucleates well on most surfaces [39,40,50,53]. Al_2_O_3_ has excellent sealing properties and has shown outstanding versatility as a barrier layer on metals [54,55,56,57,58], polymers [48,59,60,61,62], and electronics [63,64,65,66,67]. However, the use of Al_2_O_3_ as a corrosion barrier is limited by its chemical stability and dissolution in alkaline media [68,69]. On the other hand, titania is lauded for its chemical stability [68,70,71,72] and has shown favorable corrosion resistance [73,74,75,76,77,78]. Titania ALD, however, exhibits film nucleation issues using titanium tetrachloride (TiCl_4_) as a precursor, especially on copper substrates, and tends to deposit with high roughness, leading to increased porosity and poor corrosion performance over time [73,75,79].

Previous studies of ALD films for corrosion protection of copper have mostly focused on aluminum oxide. Mirhashemihaghighi et al. [80] reported a 7x increase in polarization resistance over uncoated, polished copper with a 10 nm ALD Al_2_O_3_ film and a three order of magnitude increase with a 50 nm Al_2_O_3_ film in deoxygenated 0.5 M NaCl. Chai et al. [81,82] found better corrosion protection with increasing alumina film thickness in aerated 0.1 M NaCl, as did Daubert et al. [79]. Daubert et al. [79] also performed electrochemical impedance spectroscopy (EIS) over 90 h for 5 different metal oxide ALD films, showing a decrease in high-frequency impedance for the Al_2_O_3_ film and better stability for the other four. A study by Abdulagatov et al. utilized an alumina base layer and titania capping layer to overcome the limitations of the individual layers, resulting in significantly improved corrosion protection of copper over 900 h in water at 25° and 90 °C [73]. Although not specifically related to aqueous corrosion, it is worth noting that Chang et al. successfully applied 100 nm thick ALD alumina films to copper for protection against oxidation in air at 200 °C [54]. Whereas ALD alumina possesses excellent sealing properties and can provide adequate initial corrosion protection with films less than 50 nm thick, its longer-term stability on copper is in question. Preliminary results of combining alumina and titania prove promising as a corrosion barrier, and further investigation into nanolaminate alumina/titania film structures is warranted. Reports of Al_2_O_3_/TiO_2_ nanolaminated ALD thin films for corrosion protection of steel are available [75,83,84], showing increased corrosion and delamination resistance over single-layer films.

Here we investigate the corrosion behavior of ALD-coated copper and use Al_2_O_3_/TiO_2_ nanolaminate films to enhance corrosion protection over the single-layer materials. The effect of dissolved oxygen on the corrosion protection of these ALD thin films has previously not been reported. Oxygen is always present in some amount, even when low oxygen levels are maintained in contact with the copper surfaces. Determining its impact on the protective performance of ALD films is essential for their use in RF devices and other copper-containing components. Also of interest is the effect of ALD film layer structure, copper-based interfacial films, and deposition temperature on the corrosion behavior of ALD alumina- and titania-coated copper. In this work, we utilize DC voltammetry and electrochemical impedance spectroscopy (EIS) to investigate corrosion behavior and probe the stability of Al_2_O_3_/TiO_2_ ALD films on copper in a sodium chloride (NaCl) solution with and without dissolved oxygen.

## 2. Materials and Methods

### 2.1. Material Preparation

#### 2.1.1. Substrate Preparation

Grade 110 copper sheets (99.9%, McMaster-Carr, Douglasville, GA, USA) were cut into 15 mm × 25 mm × 1 mm coupons. Coupons had a No. 8 mirror finish with average surface roughness of 0.1–0.3 µm. Prior to loading into the ALD reactor, all coupons were ultrasonically rinsed in acetone for 5 min, rinsed with isopropanol and DI water, then dipped in 35% H_3_PO_4_ for 30 s to reduce the native copper oxide, and finally rinsed in DI water and dried thoroughly with ultra-high-purity nitrogen.

#### 2.1.2. Thin Film Deposition and Characterization

Films were deposited in a home-built, viscous-flow, hot-wall ALD reactor using ultra-high-purity nitrogen (N_2_—99.999%, Arc3 Gases, Richmond, VA, USA) as the carrier and purge gas. Nitrogen was constantly flowing during deposition, maintaining a reactor pressure of roughly 1 torr. Alumina (Al_2_O_3_) was deposited using trimethylaluminum (TMA, 98% Strem Chemicals, Newburyport, MA, USA), and titania (TiO_2_) was deposited using titanium tetrachloride (TiCl_4_, 99% Strem Chemicals, Newburyport, MA, USA). DI water served as the co-reactant for both precursors. Each ALD cycle consisted of a 0.1 s precursor (TMA or TiCl_4_) dose followed by a 45 second N_2_ purge and a 0.1 s H_2_O dose followed by a 45 s N_2_ purge. Unless otherwise specified, Al_2_O_3_ and TiO_2_ were deposited at 150 °C with nominal growth rates of 1.25 Å/cycle and 0.45 Å/cycle, respectively, measured using spectroscopic ellipsometry (J.A. Wollam Co., Lincoln, NE, USA) on the copper coupons and on silicon monitor wafers coated simultaneously with the copper coupons. A Keyence VKx1100 confocal laser scanning microscope (Itasca, IL, USA) equipped with a 404 nm violet laser was used for imaging, and energy dispersive X-ray spectroscopy (EDX) was performed using a JEOL 6010LA scanning electron microscope (Peabody, MA, USA, results not shown).

### 2.2. Electrochemistry

Coated and uncoated copper coupons were placed in a 3-electrode corrosion cell (Princeton Applied Research, model K0235, Oak Ridge, TN, USA) with a platinum mesh counter electrode, a double junction Ag/AgCl reference electrode filled with 3.8 M KCl (F-600, Broadley-James, Irvine, CA, USA), and a 1 cm^2^ nominal working electrode area. All potentials given are measured against this Ag/AgCl reference. Measurements were performed at room temperature in neutral 0.1 M NaCl solution, prepared with crystalline sodium chloride (Reagent grade, Fisher Scientific, Hampton, NH, USA) and DI water, using a BioLogic VMP3 potentiostat (Seyssinet-Pariset, France). Approximately 200 mL of electrolyte was used for each test with an average initial pH of 6.7 ± 0.3. For experiments where deoxygenation was performed, nitrogen was bubbled through the electrolyte for at least 15 min prior to and continuously during testing. The nitrogen was passed through a gas washing bottle of 0.1 M NaCl to pre-saturate the dry gas before its introduction into the test cell. ‘Oxygenated’ here refers to cases in which no nitrogen purging of the electrolyte was performed.

All samples are cathodically polarized at −1.5 V for 2.5 min upon immersion in the electrolyte and prior to testing to reduce ambient or aqueously formed surface films, and all electrochemical measurements were performed at least twice to ensure repeatability.

#### 2.2.1. Electrochemical Impedance Spectroscopy

Before impedance testing, the open circuit potential was typically measured for 2 h to ensure the cell was at equilibrium, after which impedance measurements were collected at the open circuit potential over a frequency range of 100 kHz to 5 mHz at 10 frequencies per decade with two measurements per frequency and a 10 mV rms perturbation signal. Fitting impedance spectra to equivalent circuits was performed using the EC-Lab software (BioLogic, Seyssinet-Pariset, France), and all fits produced a relative error of less than 5%.

**Figure 1:**
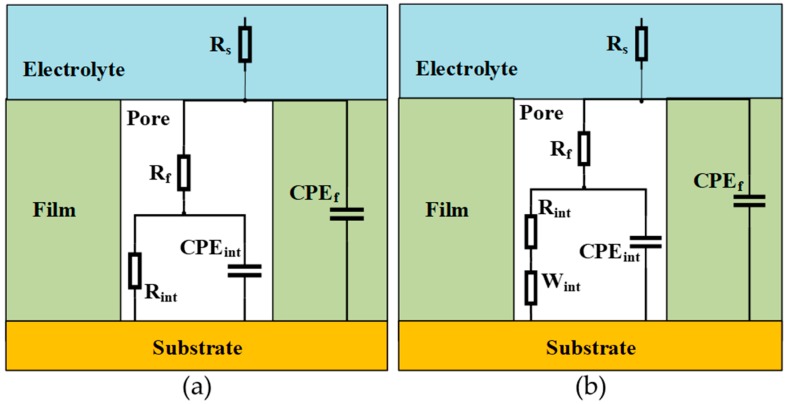
Equivalent electrical circuit used to fit impedance spectra measured in (**a**) deoxygenated and (**b**) oxygenated electrolyte. R_s_ is the solution (electrolyte) resistance, R_f_ is the film (or pore) resistance, CPE_f_ is the constant phase element representing film capacitance, CPE_int_ is the constant phase element representing interfacial capacitance (metal-solution interface), R_int_ is the interfacial resistance, and W_int_ is the interfacial Warburg diffusion element.

The equivalent electrical circuits used to fit impedance spectra are displayed in Figure 1 and are often used to model impedance data for inert coatings on metals [76,77,79,85]. The model assumes that corrosion occurs at the base of coating defects or pores, where electrolyte encounters the substrate. In this simplified model, there are parallel current paths: one through the intact coating, which is a dielectric, and the other through pores in the coating structure, represented by the film (or pore) resistance. This resistance may be viewed as the sum of the resistance of individual pores filled with electrolyte. The path through coating pores results in the substrate in contact with electrolyte, and the interfacial impedance consists of a parallel R-C circuit, where the resistance (R_int_) is typically the polarization or charge transfer resistance, and the capacitance is often the double layer capacitance. Capacitors have been replaced with constant phase elements (CPEs) to account for the frequency dispersion of the capacitance encountered in electrode-electrolyte systems [86,87,88]. The typical expression for the impedance of a CPE is given by [87,88]:

(3) $$Z_{CPE}=\frac{1}{Q{\left(j\omega \right)}^{\alpha }},$$

where *Z_CPE_* is the impedance of the constant phase element [Ω cm^2^], Q is the CPE coefficient [Ω^−1^ cm^−2^ s^α^], j is the imaginary unit [j = $\sqrt{-1}$], ω is the angular frequency [rad/s], and α is the CPE exponent [unitless].

#### 2.2.2. DC Voltammetry

Cyclic voltammetry was performed immediately following the reduction step (−1.5 V, 2.5 min) with potential scanned from −1.5 V to 0.8 V and back to −1.5 V at 10 mV/s. Additional polarization curves were measured from −1 V to 0.8 V and back to −1 V with a scan rate of 0.5 mV/s after the 2.5-min cathodic reduction with a 30-min rest period at open circuit.

Polarization resistance is determined as the slope of the V-I curve at ± 10 mV from the corrosion potential, and corrosion current is estimated using [89]:

(4) $${\frac{d\eta }{dI}|}_{\eta \to 0}=R_{p}=\frac{\beta_{a}\beta_{c}}{2.3I_{corr}\left(\beta_{a}+\beta_{c}\right)},$$

where *η* is the overpotential [V], I is the current [A cm^−2^], *R_p_* is the polarization resistance [Ω cm^2^], *I_corr_* is the corrosion current [A cm^−2^], and *β_a_* and *β_c_* are the anodic and cathodic Tafel coefficients [V/decade], respectively.

## 3. Results and Discussion

### 3.1. Spectroscopic Ellipsometry

Al_2_O_3_ and TiO_2_ ALD film thicknesses were measured using spectroscopic ellipsometry (SE) on polished copper coupons as well as silicon wafers coated simultaneously with the copper. The five coatings tested here are detailed in Table 1, along with their naming conventions and film thicknesses. Note that for all nanolaminate films (ATx1, ATx5, and ATx10), the alumina layer is deposited first (adjacent to the substrate), followed by titania. Thicknesses presented on copper were measured by spectroscopic ellipsometry (SE) after deposition and were averaged over six coupons coated together. Film thicknesses measured on copper are approximate based on a finite surface roughness and limitations in modeling of SE data, though they mostly agree with thicknesses measured on the silicon wafers. Increases in thickness above those measured on silicon may be indicative of a slightly higher growth rate for the TiCl_4_-H_2_O ALD process on copper or increased growth based on the proximity of the coupons to each other. The relative ratio of titania to alumina in the nanolaminate films is apparent in the refractive index, which was calculated from films grown on silicon. Despite differences in the number of layers, ATx10 and ATx5 have nearly identical refractive indices, confirming similar titania to alumina ratios.

### 3.2. DC Voltammetry

**Figure 2:**
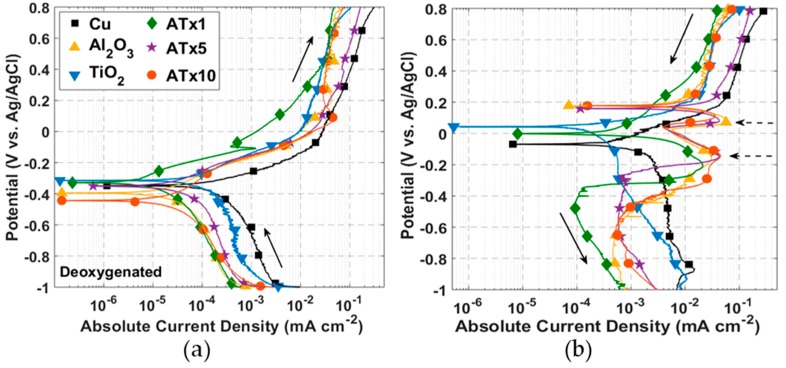
DC polarization curves: (**a**) forward scan [−1, 0.8] V and (**b**) reverse scan [0.8, −1] V measured at a scan rate of 0.5 mV/s in deoxygenated 0.1 M NaCl. Solid arrows specify scan direction and dashed arrows indicate features of interest. Refer to Table 1 for sample descriptions.

**Figure 3:**
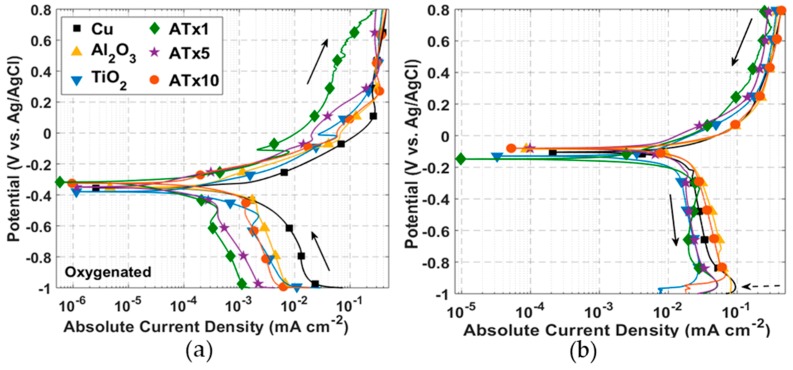
DC polarization curves: (**a**) forward scan [−1, 0.8] V and (**b**) reverse scan [0.8, −1] V measured at a scan rate of 0.5 mV/s in oxygenated 0.1 M NaCl. Solid arrows specify scan direction and dashed arrows indicate features of interest. Refer to Table 1 for sample descriptions.

ALD films were first investigated using DC voltammetry techniques. Figure 2 and Figure 3 present cyclic polarization curves measured in deoxygenated and oxygenated 0.1 M NaCl, respectively, after a 30-min rest period at open circuit following cathodic polarization. Relevant parameters determined from these curves may be found in Table 2. Coated and uncoated copper in Figure 2 and Figure 3 display similarly shaped polarization curves, suggesting analogous corrosion mechanisms consistent with electrochemically inert films. Moreover, both curves exhibit behavior that is typical of unalloyed copper in chloride media [26]. The anodic behavior includes: (i) an apparent Tafel region at low overpotentials, where Equation (1) accounts for most of the measured current; (ii) a peak current and a current reduction region, where CuCl (and possibly Cu_2_O or Cu(OH)_2_) builds up on the surface; (iii) a limited current, or passive, region where the formation and dissolution of surface films are somewhat at equilibrium; and (iv) a higher potential region where dissolution increases and oxidation of CuCl_2_^−^ to form Cu^++^ becomes significant.

#### 3.2.1. Deoxygenated Electrolyte

TiO_2_ and ATx1 exhibit an anodic shift in corrosion potential from the uncoated copper. This has been attributed to modification of the substrate exposed through coating defects [55], as well as native oxide formation during ALD growth and aging of the oxide during storage [80]. The remaining corrosion potentials are shifted cathodically, a trend that has also been reported for ALD coatings on stainless steel [75,84], copper [79], and a Mg-Al alloy [83]. All coated coupons show an increase in polarization resistance and decrease in corrosion current compared to uncoated copper. Polarization resistance is better than an order of magnitude higher for the nanolaminate Al_2_O_3_/TiO_2_ films.

**Figure 4:**
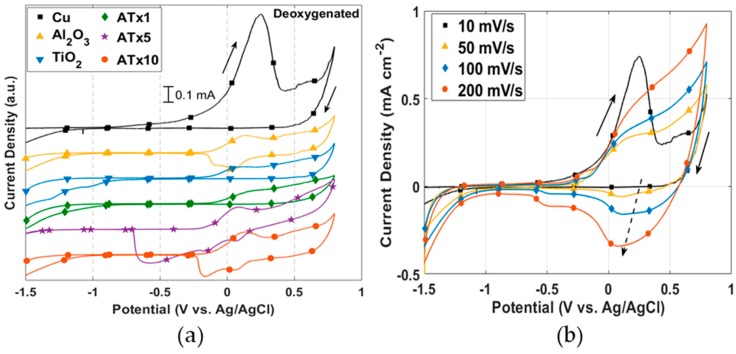
I–V curves in deoxygenated 0.1 M NaCl for: (**a**) uncoated and ALD-coated copper measured at 10 mV/s and (**b**) uncoated copper measured at scan rates from 10 mV/s to 200 mV/s. Solid arrows indicate scan direction and dashed arrow in (**b**) points in the direction of increasing scan rate. Refer to Table 1 for sample descriptions.

The current peak associated with CuCl film buildup is apparent in Figure 4a, measured with a voltage scan rate of 10 mV/s. This suggests that the kinetics of the reaction leading to the precipitation of CuCl (Equation (1)) are faster than can be observed with a 0.5 mV/s scan rate. This is at least true for the uncoated copper. A current peak is visible in Figure 2a for several of the ALD-coated samples, likely corresponding to CuCl buildup at exposed copper surface sites. The peaks in the forward scan of Figure 4a are not as pronounced for coated copper as for the uncoated copper, showing that the coatings restrict the amount of surface area that is exposed to electrolyte.

Current measured over the forward scan is as much as an order of magnitude smaller for ALD-coated compared to uncoated copper. The curves never quite merge, though the difference in current decreases above ~0.4 V, as the CuCl surface coverage reaches a maximum at the Cu-ALD film interface. The reverse potential scans in Figure 2b show two large current peaks for Al_2_O_3_, ATx5, and ATx10 at ~ 0.1 V and -0.15 V. Only one of these peaks is seen for ATx1, and they do not appear at all in the curves for TiO_2_ and uncoated copper. These current peaks are likely associated with copper-based film reduction at the Cu-ALD coating interface. Figure 4b shows that the presence of this reduction peak for uncoated copper is dependent on potential scan rate. This feature appears when the potential is scanned at 50 mV/s and becomes more prominent at 100 and 200 mV/s. The resistance of pores in some of the ALD films clearly slows this reduction to the point that the peaks are visible at low scan rates.

#### 3.2.2. Oxygenated Electrolyte

Again, all coated samples demonstrate higher polarization resistance and lower corrosion current, and most have anodically-shifted corrosion potentials (see Figure 3). Several samples have similar corrosion potentials to the deoxygenated electrolyte, though all polarization resistances are smaller than their deoxygenated counterparts. Because dissolved oxygen alone is not typically enough to passivate the copper surface, the oxygen content should have little effect on the equilibrium potential. The larger differences in corrosion potential between oxygenated and deoxygenated conditions for Al_2_O_3_, TiO_2_, and ATx10 may be attributed to slight differences in surface conditions, post-deposition aging, or film quality.

There is evidence of diffusion-limited current in the cathodic portion of the curves in Figure 3. In this case, the polarization resistance cannot be taken as a direct measurement of the charge transfer resistance, and thus the corrosion current should be considered approximate. Regardless of the diffusion-limited cathodic current, anodic dissolution of copper in chloride media is known to be under mixed charge transfer and mass transport control [31], so care should always be taken in interpreting corrosion parameters from polarization curves. In this case, the polarization resistance and corrosion current serve as adequate comparison points between samples.

Cathodic currents measured during the forward potential scan are all smaller for the ALD-coated copper, indicating a smaller exposed area for oxygen reduction at the copper surface. Measured current for most samples converges once passivation occurs above 0.1–0.3 V. The shape of the reverse scans (Figure 3b) is nearly identical for all samples, and a current peak appears close to −1 V associated with reduction of the Cu_2_O surface film formed during the forward scan.

### 3.3. Electrochemical Impedance Spectroscopy

**Figure 5:**
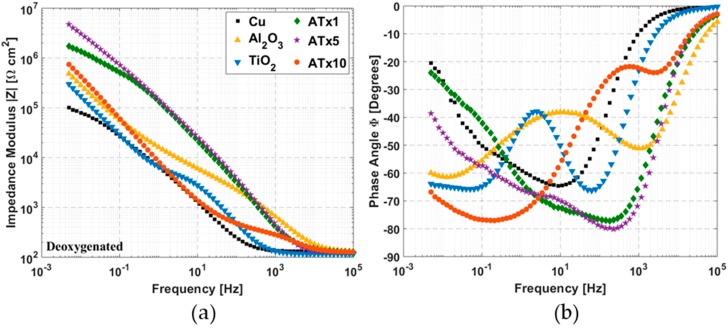
Impedance spectra measured from 100 kHz to 5 mHz in deoxygenated 0.1 M NaCl at OCP: (**a**) impedance modulus and (**b**) phase angle versus frequency. Refer to Table 1 for sample descriptions.

**Figure 6:**
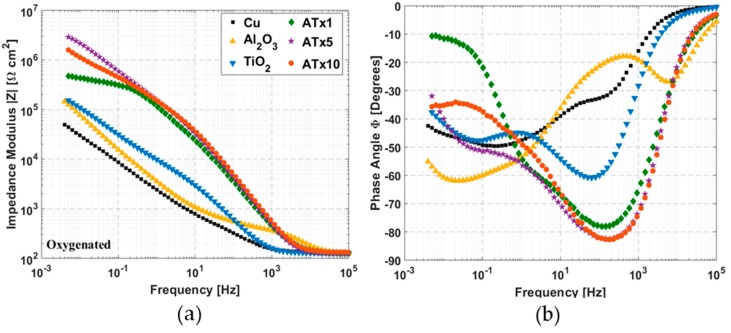
Impedance spectra measured from 100 kHz to 5 mHz in oxygenated 0.1 M NaCl at OCP: (**a**) impedance modulus and (**b**) phase angle versus frequency. Refer to Table 1 for sample descriptions.

Figure 5 and Figure 6 show impedance spectra of coated and uncoated copper in deoxygenated and oxygenated 0.1 M NaCl, respectively. The graphs are split into Bode plots of impedance modulus and phase angle versus frequency for clarity. All impedance spectra were collected at open circuit potential, and the results of equivalent circuit modeling of impedance data are provided in Table 3.

#### 3.3.1. Deoxygenated Electrolyte

ALD films exhibit increases in impedance over uncoated copper, generally 1–2 orders of magnitude at low frequency. The phase angle plot depicts differences in the high-frequency impedance response associated with the ALD films. Nanolaminate films have the largest impedance increase, though all the ALD films have distinct impedance response from the uncoated copper sample.

There are several things of note in Table 3. The film resistance of ATx1 and ATx5 is much higher than the other ALD coatings. Also, the interfacial CPE exponent is quite low for several of the samples, indicating significant capacitive dispersion at the underlying copper surface. This may partly explain the disagreement between interfacial resistance values from EIS and polarization resistances from DC voltammetry (Table 2), as well as the effect of having an insufficiently low cutoff frequency. However, the fact that the EIS-derived polarization resistances are higher is most likely influenced by the longer rest period following reduction before impedance spectra are recorded. The impact of rest time and aging is explored in Section 3.7. Equivalent circuit modeling reveals that several of the ALD films exhibit CPE exponents above 0.9, indicating high-quality coatings, and fitted interfacial resistances for all coatings are higher than the uncoated copper.

#### 3.3.2. Oxygenated Electrolyte

The nanolaminate films show increases in impedance as high as two orders of magnitude. The equivalent circuit for oxygenated measurements (Figure 1b) contains an additional Warburg diffusion element at the copper-ALD film interface to account for enhanced mass transport control in the presence of dissolved oxygen. Data were only fit well with the Warburg element in two cases, ATx1 and ATx10. For other samples the fitted Warburg coefficient was negligible. Film resistances for the nanolaminate ALD films are much higher than the single layers and the deoxygenated measurements, indicating passivation of pores in the more complex film structures. Interfacial resistances for all coated samples are higher than the bare copper, although they are lower than those determined from deoxygenated measurements, as would be expected.

### 3.4. ALD Film Stability

**Figure 7:**
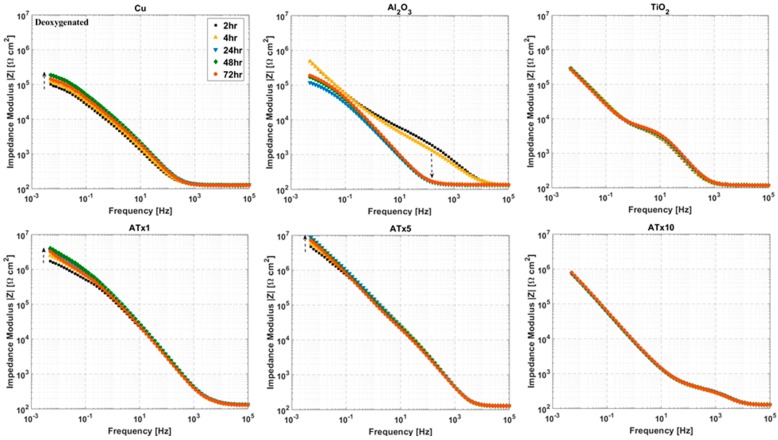
Impedance modulus versus frequency at selected time intervals over 72-h immersion in deoxygenated 0.1 M NaCl. Frequency range is 100 kHz to 5 mHz and sample type is given above each plot. Dashed arrows indicate increasing immersion time. Refer to Table 1 for sample descriptions.

**Figure 8:**
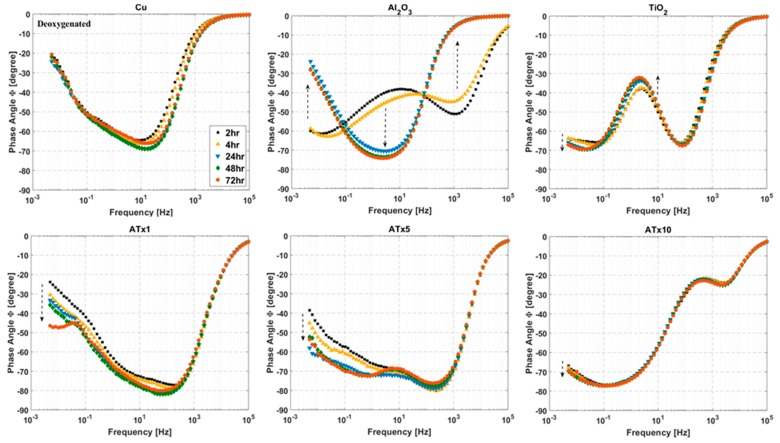
Impedance phase angle versus frequency at selected time intervals over 72-h immersion in deoxygenated 0.1 M NaCl. Frequency range is 100 kHz to 5 mHz and sample type is given above each plot. Dashed arrows indicate increasing immersion time. Refer to Table 1 for sample descriptions.

**Figure 9:**
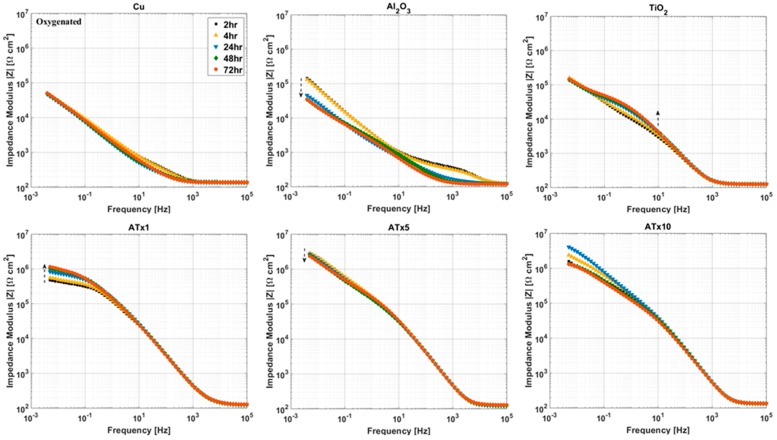
Impedance modulus versus frequency at selected immersion times over 72-h immersion in oxygenated 0.1 M NaCl. Frequency range is 100 kHz to 5 mHz and sample type is given above each plot. Dashed arrows indicate increasing immersion time. Refer to Table 1 for sample descriptions.

**Figure 10:**
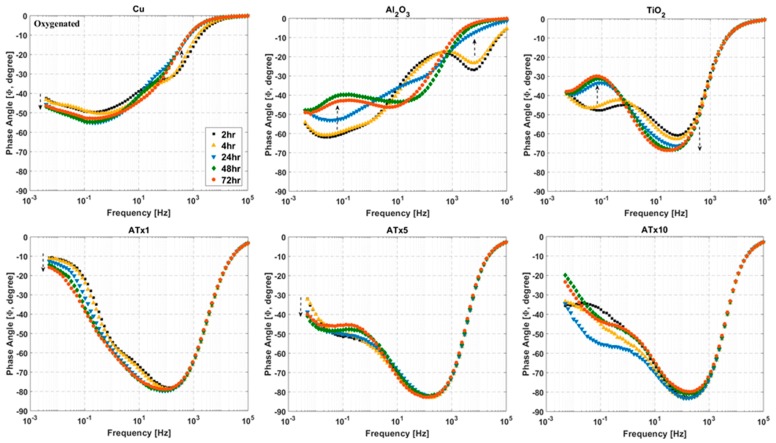
Impedance phase angle versus frequency at selected immersion times over 72-h immersion in oxygenated 0.1 M NaCl. Frequency range is 100 kHz to 5 mHz and sample type is given above each plot. Dashed arrows indicate increasing immersion time. Refer to Table 1 for sample descriptions.

**Figure 11:**
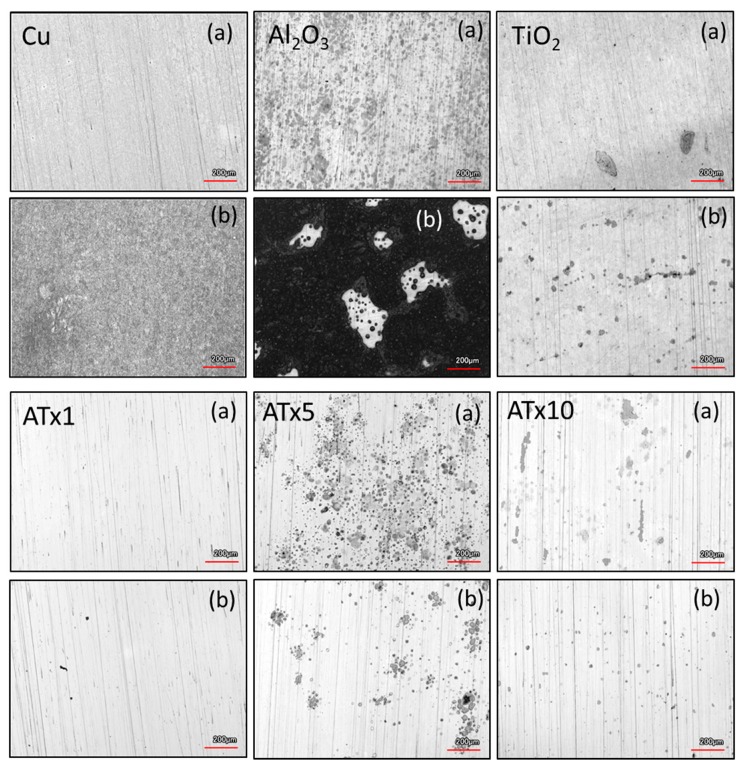
Confocal laser scanning microscope images of uncoated and ALD-coated copper samples after 72-h immersion and EIS testing in (**a**) deoxygenated and (**b**) oxygenated 0.1 M NaCl. Refer to Table 1 for sample descriptions.

Impedance spectra measured over a 72-h immersion in deoxygenated and oxygenated electrolyte are presented in Figure 7, Figure 8, Figure 9 and Figure 10. Again, the graphs are split into Bode plots of impedance modulus and phase angle versus frequency, and measurements were made at open circuit conditions. Dashed arrows are provided in some of the graphs to indicate an increase in immersion time. Laser scanning microscope images of coated and uncoated copper after 72-h EIS testing are displayed in Figure 11.

#### 3.4.1. Deoxygenated Electrolyte

Phase angle plots show differences in impedance response among the ALD films and highlight changes in the samples over time. Uncoated copper undergoes an increase in impedance, peaking at 24–48 h. This is due to the buildup of a protective surface film (i.e., CuCl). ATx5 and ATx1 also exhibit similar behavior. Variations in phase angle at low frequency for these two films indicate changes at the ALD film-substrate interface, rather than much alteration in the film itself. TiO_2_ and ATx10 display good stability over 72 h, with minimal deviation in modulus or phase angle visible. A change in the Al_2_O_3_ sample over time is apparent in Figure 7 and Figure 8. The change in shape of both the modulus and phase angle spectra indicate degradation of the ALD alumina film. The increase in phase angle and decrease in impedance at high frequency seen after 24 h point to a stark decrease in the dielectric quality of the film. Indeed, the shape of these curves for Al_2_O_3_ after 24 h closely resemble those of the bare copper, and the impedance of the Al_2_O_3_ sample drops below that of uncoated copper due to severe ALD film damage.

Laser scanning microscope images after 72 h of EIS testing reveal that the uncoated copper surface remains smooth and generally featureless after 72 h, having undergone uniform corrosion along with Cu-based surface film buildup and dissolution during the immersion period. TiO_2_ and ATx1 remain mostly unchanged, in good agreement with the impedance spectra. ATx5 and ATx10 show signs of corrosion spots, though the ALD films are still intact, which also agrees with EIS measurements over 72 h. In the case of ATx5, these appear to be superficial spots of discoloration rather than corrosion of the underlying copper surface. Al_2_O_3_ seems to be somewhat degraded, as the smoothness of the substrate is apparently gone. Only a trace amount of aluminum is detected using EDX (not shown) after immersion and is approximately 2% of what was detected before exposure to the NaCl solution. An explanation of the degradation of the alumina ALD film is presented in Section 3.5.

#### 3.4.2. Oxygenated Electrolyte

The titania-containing films have good stability over the immersion time, with TiO_2_, ATx1, and ATx10 having increases in low-frequency impedance over time due to passivation of the substrate. Al_2_O_3_ again undergoes degradation, evidenced by decreasing impedance modulus in a frequency range corresponding to film response, which is mirrored by variations in shape of the phase angle over time. The change in impedance of the Al_2_O_3_ film makes the degradation seem less severe than in deoxygenated conditions, though the laser scanning microscope image in Figure 11 shows what appears to be worse damage. However, the aluminum content detected by EDX (not shown) after immersion is approximately 25% of what is detected before testing, which is 10 times more than that detected after exposure to deoxygenated NaCl. The high contrast in the Al_2_O_3_ image in Figure 11 shows regions of exposed copper surrounded by dark, rough patches. The substrate has clearly been oxidized in most places, likely slowing the deterioration of the remaining Al_2_O_3_ ALD film. Other ALD samples appear to be in good shape, and the laser images are similar to those taken after immersion in deoxygenated electrolyte.

### 3.5. Dissolution of Al_2_O_3_

According to thermodynamics, alumina should be stable in neutral aqueous electrolyte [69]. However, it has been shown in several instances that ALD alumina films experience dissolution in neutral NaCl solutions [76,90]. Diaz et al. proposed that this behavior for ALD Al_2_O_3_ on carbon steel resulted from cathodic oxygen reduction at the substrate surface, accessible through defects in the ALD film [90]. Recall that oxygen reduction proceeds through the first reaction in Equation (2). This reaction generates hydroxide ions, leading to a localized increase in pH and facilitating dissolution according to [90]:

(5) $$Al_{2}O_{3}+2OH^{-}\to 2AlO_{2}^{-}+H_{2}O$$

**Figure 12:**
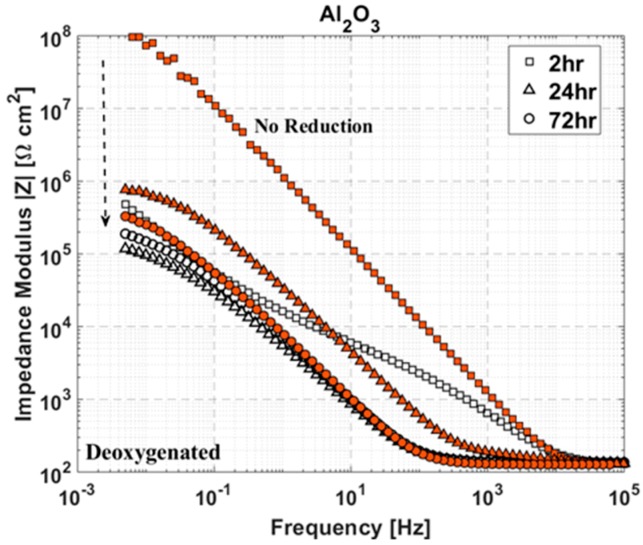
Impedance modulus versus frequency at selected immersion times over 72 h in deoxygenated 0.1 M NaCl for ALD Al_2_O_3_ on copper. Unfilled shapes (black) are with and filled shapes (red) are without an initial 2.5-min reduction at −1.5 V. Dashed arrow points in the direction of increasing immersion time.

The degradation of ALD Al_2_O_3_ on copper over 72 h in 0.1 M NaCl seen in this work supports the dissolution of alumina in neutral electrolyte. As in the work of Diaz et al. [90], deterioration of the alumina film took place in deoxygenated electrolyte as well as oxygenated. This suggests either incomplete removal of dissolved oxygen, coupled with a very low threshold for oxygen reduction to occur, or the generation of hydroxide ions through other means, possibly water reduction (see Equation (2)). Both scenarios likely contribute: incomplete removal of dissolved oxygen has been proposed in the past [80] and some water reduction should occur at the high cathodic overpotentials to which the samples are exposed. However, even in the absence of the reduction step (at −1.5 V) the ALD alumina film degrades similarly according to EIS spectra, displayed in Figure 12. Thus, hydroxide ions generated at equilibrium conditions must be sufficient to cause dissolution, assuming Equation (5) is the primary reaction pathway for alumina ALD films.

A recent study investigated the stability of ALD alumina films on silicon substrates in aqueous media at elevated temperatures [68]. This study did not report dissolution of alumina in neutral electrolyte, which is not surprising since oxygen reduction would likely not occur at the Si surface to generate the required hydroxide ions, according to Equation (5). However, the authors did show hydration and swelling, followed by film restructuring and roughening during exposure to neutral DI water at room temperature with and without chloride ions. Alumina is known to exist in its hydrated form when in contact with water [69]. Swelling and roughening would increase porosity and exposed surface area, which could enhance the overall degradation of the alumina-coated copper. Note that the hydration and dissolution mechanisms discussed here are not mutually exclusive and could both contribute to the observed phenomena.

### 3.6. Effect of Dissolved Oxygen

The presence of dissolved oxygen is known to shift the equilibrium of Equation (1) toward CuCl_2_^−^, leading to a higher dissolution rate with higher oxygen content [28]. The enhanced dissolution of CuCl does not allow it to reach maximum coverage on the surface and inhibits passivation until a critical current of 0.3–0.4 mA cm^−2^ is reached. The passivation current is more variable in deoxygenated electrolyte, though it is roughly an order of magnitude smaller than in the presence of oxygen. Reverse potential scans with oxygen present (Figure 3b) are significantly less variable than in deoxygenated solutions. This, coupled with the single reduction peak for all samples, indicates a distinct mechanism from deoxygenated media, which is presumed to be the formation of a Cu_2_O surface film. The reduction of this film appears to be a slower reaction, as this peak is evident at a scan rate of 0.5 mV/s, whereas the CuCl reduction on the bare copper was not visible until scan rates of 50–100 mV/s were used.

**Figure 13:**
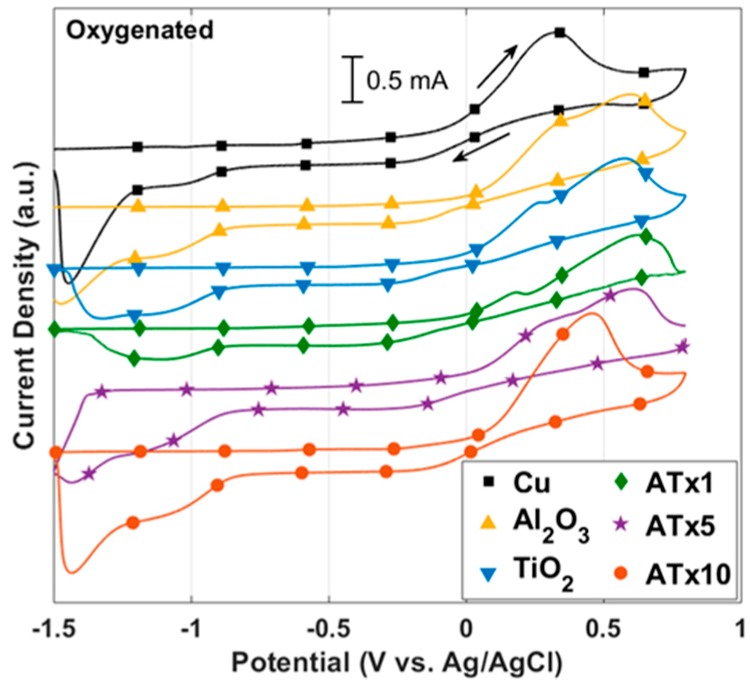
I–V curves in oxygenated 0.1 M NaCl for coated and uncoated copper, measured at a scan rate of 10 mV/s. Arrows indicate the scan direction.

There is a current peak and subsequent reduction in the anodic portion of the polarization curves for some of the ALD samples in oxygenated NaCl. This additional current peak is more obvious in the CV scans provided in Figure 13 and generally results in a broadening of the passivation peak. This may be indicative of maximum CuCl coverage at the Cu surface sites exposed through coating defects. The much smaller substrate area exposed through pores in the ALD films compared to the uncoated copper could promote full CuCl surface coverage before full oxide film formation occurs.

ALD film stability is rather unaffected by dissolved oxygen in the electrolyte. Formation of a copper oxide film at the substrate-ALD film interface likely counteracts the more aggressive dissolution of copper with oxygen present. This is an important point for implementation of ALD coatings in RF devices for corrosion protection, as it is common for dissolved oxygen levels to be above specification for coolant supplies.

### 3.7. Copper-Based Surface Films and Sample Aging

Voltammetric curves were measured with and without (results not shown) a 30-min rest period to investigate the effect of copper-based surface films on electrode polarization. Samples were held at −1.5 V for 2.5 min to reduce surface films, after which either the polarization scan immediately begins or there is a 30-min rest period at the open circuit potential. In both cases the scan starts at −1 V vs. Ag/AgCl. Uncoated copper exhibits higher polarization resistance (as much as 5×) without the rest period regardless of dissolved oxygen content in the electrolyte. Both CuCl and Cu_2_O are thought to precipitate on the Cu surface during immersion in Cl-containing electrolyte [30,31,91]. The presence of these corrosion products formed during the open circuit measurement could account for the increase in cathodic currents, as copper corrosion products are known to complicate (and perhaps enhance) oxygen reduction [92]. Differences in polarization resistance and anodic current (deoxygenated electrolyte) may be indicative of patchy CuCl/Cu_2_O film formation at OCP, resulting in enhanced dissolution, rather than passivation.

The effects of a rest period after electroreduction are mixed for the ALD-coated copper samples. Polarization resistance generally decreases for ALD samples without the 30-min OCP period. This is due to a likely (partial) passivation of the copper surface at ALD film defect sites during the OCP measurement. The much smaller exposed area through pores in the ALD film compared with the uncoated copper allows surface films to precipitate faster. This is supported by the fact that the TiO_2_ sample sees an increase in polarization resistance comparable to uncoated copper. The TiO_2_ possesses inferior sealing and nucleation properties compared with Al_2_O_3_, resulting in increased porosity and allowing for a larger area of exposed Cu. In some cases, the polarization behavior is largely unchanged with or without the rest period.

Assuming we may treat the ALD films as electrochemically inert simplifies this analysis considerably. In that case, changes in polarization behavior must be ascribed to changes in the state of the copper surface or to blocking effects by the ALD films. However, the dissolution of Al_2_O_3_ would complicate this process (refer to Section 3.5). A lack of passivation of surface sites during the immediate transition from −1.5 V to −1 V for the polarization scan allows for enhanced oxygen reduction and local increases in pH. This would lead to dissolution of alumina via Equation (5), contributing to the measured current and the apparent decrease in corrosion protection.

Aging of the ALD-coated samples plays an important role in parameters derived from electrochemical testing, as is apparent from the discussion of the effect of copper surface films formed during testing. After the coupons are coated (and during deposition [93]), copper oxide growth is expected at the base of coating pores and defects. Passivation in this manner can greatly increase initial corrosion resistance. This is evident in the work of Mirhashemihaghighi et al. [80,93], in which polarization resistances on the order of 100 MΩ cm^2^ are measured without an electroreduction step during immersion in 0.5 M NaCl. Also, this is apparent in Figure 12, where the lack of a reduction step leads to a two order of magnitude increase in impedance of Al_2_O_3_-coated copper. It does not, however, reduce or prevent the dissolution of alumina over time. Further investigation is needed to determine the effect of aging of ALD films on copper on the long-term stability of the films.

### 3.8. Effect of Deposition Temperature

It is well known that many thermal ALD processes can proceed over a wide temperature range. For example, Al_2_O_3_ has been deposited by ALD from room temperature [45,94] up to 400 °C [76]. The substrate temperature during deposition can have a substantial impact on film properties, though its effect on the corrosion resistance of the film is not immediately apparent. Increasing temperature has two competing consequences for thin film growth: densification and crystallization. Higher deposition temperature for inorganic films almost always leads to an increase in density, which is desirable for corrosion protection. Along with an increase in density, certain materials begin to crystallize. Though crystallinity is preferred in many applications, amorphous corrosion barrier films are generally more effective. Crystalline films tend to have aligned pores and thus provide inferior separation between substrate and environment.

**Figure 14:**
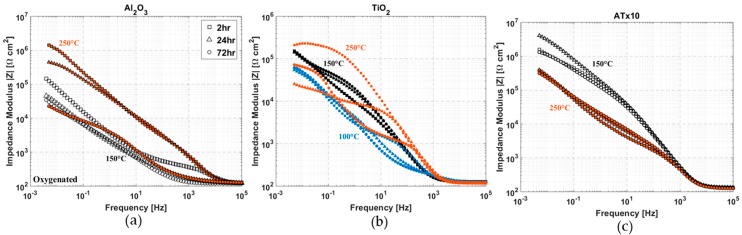
Impedance modulus versus frequency at selected immersion times over 72 h in oxygenated 0.1 M NaCl for various ALD substrate temperatures: (**a**) Al_2_O_3_-coated Cu; (**b**) TiO_2_-coated Cu; and (**c**) ATx10 [(Al_2_O_3_+TiO_2_)x10]-coated Cu. In (**a**) and (**c**) unfilled shapes (black) represent a substrate temperature of 150 °C during deposition and filled shapes (red) indicate 250 °C substrate temperature. In (**b**) substrate temperatures are: 250 °C—red, 150 °C—black, and 100 °C—blue. Deposition temperatures are also provided on each plot, and all films are nominally 50 nm thick.

ALD Al_2_O_3_ is known to be amorphous up to temperatures well beyond the upper limit of atomic layer growth [95,96], which is important for its use as a corrosion barrier coating. Higher deposition temperature leads to a denser film and decreases impurities such as hydrogen and carbon [76,97]. Figure 14a shows that this does not translate into an increase in the stability of ALD alumina on copper. Despite the higher starting film quality of the 250 °C deposition, resulting in impedance that is as much as an order of magnitude higher than the film deposited at 150°C, the stability is still poor. The 250 °C Al_2_O_3_ film experiences a 100x decrease in low-frequency impedance after 72 h, which is ~10x that of the 150 °C film. This result agrees with a previous study of ALD alumina films, in which increased chemical stability was not observed until annealing at 900 °C was performed [68].

The temperature dependence of TiO_2_ deposition is a bit more complicated. Refractive index and impurity concentrations generally increase and decrease, respectively, with increasing deposition temperature [76,98,99]. TiO_2_ ALD films deposited using the TiCl_4_-H_2_O process have been shown to be amorphous at temperatures ≤ 150 °C and crystalline above roughly 165 °C [76,98,100]. Studies have also presented film thickness [100,101] and substrate [102] dependence of TiO_2_ crystallinity by ALD. There are competing mechanisms with TiO_2_ ALD growth: higher deposition temperatures produce better film quality but also result in varying degrees of crystallization. This effect is demonstrated in Figure 14b, which shows impedance spectra for three different deposition temperatures of TiO_2_ ALD growth on copper. The films deposited at 100 °C and 150 °C both show good stability over 72 h, though the higher film quality of the 150 °C film is apparent based on the larger measured impedance from ~100 Hz to 5 mHz. The titania film deposited at 250 °C exhibits vastly different impedance spectra after 2, 24, and 72 h immersion. Unlike alumina, the chemical stability of titania in neutral salts is not in question, so this behavior is indicative of changes in the copper substrate and may be attributed to the higher porosity of this coating due to its crystallinity.

A deposition temperature of 250 °C for the 10x nanolaminate (ATx10) results in lower overall impedance (Figure 14c), though there is very little change in impedance over 72 h immersion. This contrasts the poor stability seen for alumina and titania individually at 250 °C. It is presumed that the multilayered structure generates discontinuous pores and can reduce the negative effects of the crystallization of TiO_2_ layers. It is also likely that the TiO_2_ layer (~2.5 nm) is not thick enough to become crystalline in the ATx10 film structure [100,101]. Further investigation is needed to fully understand the effects of deposition conditions on ultra-thin ALD nanolaminate film layers.

### 3.9. Nanolaminate Coatings

The effects of having a conformal sealing layer are apparent in the increased initial corrosion protection afforded by the alumina-containing coatings over the single-layer TiO_2_ film. However, due to the dissolution of alumina, this beneficial effect may be partially overshadowed. Each of the three nanolaminate coatings was a superior corrosion barrier to either of the individual materials. It was expected that increasing the number of film layers would lead to lower porosity and a better corrosion barrier, though ATx5 [(5 nm Al_2_O_3_ + 5 nm TiO_2_)x5], rather than ATx10 [(2.5 nm Al_2_O_3_ + 2.5 nm TiO_2_)x10], provided the best overall corrosion protection, as determined by EIS measurements over 72 h with and without deoxygenation. We attribute this to achieving a balance between the number of layers (and interfaces) and better film quality as thickness increases. Having multiple film layers likely facilitates the filling of pores in previous layers and the misalignment of pores across several interfaces. On the other hand, film density and uniformity generally increase with thickness for ultra-thin films. Thus, having fewer interfaces in ATx5 is balanced by generating higher-quality individual layers at 5 nm compared to 2.5 nm for ATx10. Nonetheless, the nanolaminate films successfully incorporate desirable properties of both film materials. The possibility of including additional materials, metal oxides or otherwise, allows these films to be tailorable to specific situations or environments. The precise thickness control of ALD enables these types of nanolaminate films with many individual layers, even when the total film thickness is only 50 nm or less.

## 4. Conclusions

Atomic layer deposition presents the opportunity to apply nanometer-scale corrosion barrier coatings to very high aspect ratio structures and component interiors, which is not possible with many other coating techniques. The results presented here show the promise of ultra-thin ALD barrier coatings in chloride environments. DC voltammetry measurements produced 10–15x increases in polarization resistance, up to 3.4 MΩ cm^2^, with 50 nm nanolaminate films of Al_2_O_3_ and TiO_2_, two materials that are readily available and widely deposited by ALD. With longer equilibration time than for the DC measurements, the nanolaminate films saw nearly a 100x increase in the polarization resistance determined from equivalent circuit fitting of impedance spectra, up to 12 MΩ cm^2^. This was even higher, well above 100 MΩ cm^2^, when the initial electroreduction step was removed and could be further increased using thicker films or different materials.

The stability of single-layer Al_2_O_3_ ALD films on copper was found to be poor during immersion in neutral 0.1 M NaCl, with severe degradation in measured impedance response occurring within 24 h. This agrees with previous reports of the dissolution of ALD alumina on active substrates [76,90] and has been attributed to hydroxide generation during oxygen reduction at the substrate surface exposed through pores or defects in the ALD film [90]. TiO_2_ was found to be very stable, although it showed the smallest initial increase in corrosion resistance because of its inferior nucleation and sealing capabilities compared to alumina. Nanolaminate film structures, including ATx1 [10 nm Al_2_O_3_ + 40 nm TiO_2_], ATx5 [(5 nm Al_2_O_3_ + 5 nm TiO_2_)x5], and ATx10 [(2.5 nm Al_2_O_3_ + 2.5 nm TiO_2_)x10], were successful at combining the superior sealing properties of alumina with the excellent chemical stability of titania. ATx5 exhibited the best overall performance according to impedance spectroscopy over 72 h. We attribute this to the beneficial effects of multiple thin film layers without individual layer quality suffering from being too thin to form a dense and effective barrier. It remains to be seen how these samples perform over longer immersion times and in flowing electrolyte conditions.

The effect of dissolved oxygen in the electrolyte was probed through various electrochemical measurements. As expected, the presence of oxygen enhances corrosion in chloride-containing media. However, perhaps unexpectedly, dissolved oxygen was found to have little effect on the stability of ALD films. The ability of copper exposed through coating pores to passivate caused there to be minimal variation in impedance over 72 h, comparable to deoxygenated conditions. Laser microscope imaging revealed little difference in surface morphology between nanolaminate samples exposed to oxygenated or deoxygenated electrolyte.

Also explored was the use of different deposition temperatures to determine its effect on film quality and corrosion barrier efficacy. An obviously higher initial film quality existed for Al_2_O_3_ single layers deposited at 250 °C, though degradation over time was found to be at least as, if not more, severe than for the films deposited at 150 °C, according to impedance spectra. Substrate temperature during deposition of TiO_2_ is more complicated in that it impacts the crystallinity as well as the dielectric quality and contaminant concentrations. As with alumina, the initial dielectric quality of the titania films was found to increase with deposition temperature, although crystallization and increased porosity at 250 °C resulted in poor performance over the 72-h immersion. The stability of TiO_2_ at 100 °C was good, but it was still outperformed by the 150 °C deposition, which appears to be in the range of an optimal temperature for titania ALD for corrosion protection. ATx10 was also deposited at 250 °C and showed good stability over 72 h, unlike both the single-layer alumina and titania deposited at this temperature.

## Figures and Tables

**Table 1:** Samples tested in this work—naming convention, film type, and ellipsometric data. The Si Thickness and Cu Thickness columns indicate fitted film thicknesses on the respective substrates using spectroscopic ellipsometry. Refractive indices are determined from film measurements on silicon substrates.

Sample Name	Nominal Film Structure	Si Thickness (nm)	Cu Thickness (nm)	Refractive Index
Cu	None—uncoated copper	-	-	-
Al_2_O_3_	Single-layer Al_2_O_3_	52.9 ± 0.6	49.1 ± 0.7	1.64
TiO_2_	Single-layer TiO_2_	54.0 ± 1.6	63.8 ± 2.8	2.43
ATx10	Nanolaminate—(Al_2_O_3_ + TiO_2_) × 10	56.0 ± 0.6	65.8 ± 2.5	2.1
ATx5	Nanolaminate—(Al_2_O_3_ + TiO_2_) × 5	52.5 ± 0.8	56.6 ± 1.9	2.12
ATx1	Double layer—10 nm Al_2_O_3_ + 40 nm TiO_2_	52.6 ± 0.9	60.9 ± 1.1	2.33

**Table 2:** Corrosion potential (E_corr_), polarization resistance (R_p_), and corrosion current (I_corr_) determined from polarization curves found in deoxygenated (Figure 2) and oxygenated (Figure 3) 0.1 M NaCl.

Sample	Deoxygenated			Oxygenated		
	E_corr_ (V vs. Ag/AgCl)	R_p_ (Ω cm^2^) × 10^6^	I_corr_ (A cm^−2^) × 10^−7^	E_corr_ (V vs. Ag/AgCl)	R_p_ (Ω cm^2^) × 10^6^	I_corr_ (A cm^−2^) × 10^−7^
Cu	−0.347	0.191	1.51	−0.355	0.066	3.77
Al_2_O_3_	−0.395	1.67	0.179	−0.314	0.204	1.07
TiO_2_	−0.314	0.373	0.915	−0.379	0.193	0.992
ATx1	−0.328	3.37	0.103	−0.318	0.948	0.167
ATx5	−0.350	2.60	0.115	−0.349	0.596	0.340
ATx10	−0.444	1.85	0.197	−0.325	0.167	1.09

**Table 3:** Fitted parameters from equivalent electrical circuit modeling of impedance spectra in deoxygenated (Figure 5) and oxygenated (Figure 6) 0.1 M NaCl. Refer to Figure 1 for a depiction of the equivalent circuit and parameter definitions. Q and α are the coefficient and exponent of the constant phase element (see Equation (3)).

**Deoxygenated**								
	**R_s_** **(Ω cm^2^)**	**Q_film_** **(Ω^−1^ s^a^ cm^−2^)**	**α_film_**	**R_film_** **(Ω cm^2^)**	**Q_int_** **(Ω^−1^ s^a^ cm^−2^)**	**α_int_**	**R_int_** **(Ω cm^2^)**	
**Cu**	124.7	2.08 × 10^−5^	0.87	6831	3.14 × 10^−5^	0.62	1.23 × 10^5^	
**Al_2_O_3_**	120.1	2.65 × 10^−6^	0.74	5320	2.04 × 10^−5^	0.65	6.30 × 10^6^	
**TiO_2_**	116.5	5.63 × 10^−6^	0.93	4759	4.83 × 10^−5^	0.80	1.48 × 10^6^	
**ATx1**	130.2	9.05 × 10^−7^	0.91	11,642	1.54 × 10^−6^	0.4	3.97 × 10^6^	
**ATx5**	126.2	4.43 × 10^−7^	0.98	18,858	1.45 × 10^−6^	0.63	1.11 × 10^7^	
**ATx10**	126.3	3.75 × 10^v6^	0.79	343.5	2.08 × 10^−5^	0.86	5.19 × 10^6^	
**Oxygenated**								
	**R_s_** **(Ω cm^2^)**	**Q_film_** **(Ω^−1^ s^a^ cm^−2^)**	**α_film_**	**R_film_** **(Ω cm^2^)**	**Q_int_** **(Ω^−1^ s^a^ cm^−2^)**	**α_int_**	**R_int_** **(Ω cm^2^)**	**W_int_** **(Ω s^−0.5^ cm^2^)**
**Cu**	135.6	8.08 × 10^−5^	0.90	272.5	1.46 × 10^−4^	0.58	2.18 × 10^5^	2.2 × 10^−9^
**Al_2_O_3_**	116.8	3.02 × 10^−6^	0.74	385	8.96 × 10^−5^	0.73	7.60 × 10^5^	4.8 × 10^−10^
**TiO_2_**	126	7.16 × 10^−6^	0.86	5324	3.81 × 10^−5^	0.58	4.85 × 10^5^	9.2 × 10^−10^
**ATx1**	128	8.03 × 10^−7^	0.91	65,445	1.26 × 10^−6^	0.77	3.02 × 10^5^	1.6 × 10^4^
**ATx5**	123.9	4.58 × 10^−7^	0.97	72,013	1.96 × 10^−6^	0.64	5.50 × 10^6^	1.6 × 10^−3^
**ATx10**	134.3	3.35 × 10^−7^	0.99	24,467	2.63 × 10^−6^	0.42	4.58 × 10^6^	4.4 × 10^5^
